# A review of nanomaterials from synthetic and natural molecules for prospective breast cancer nanotherapy

**DOI:** 10.3389/fphar.2023.1149554

**Published:** 2023-05-19

**Authors:** Pankaj Kumar, Bharti Mangla, Shamama Javed, Waquar Ahsan, Pankaj Musyuni, Durgaramani Sivadasan, Saad S. Alqahtani, Geeta Aggarwal

**Affiliations:** ^1^ Centre For Advanced Formulation and Technology, Delhi Pharmaceutical Sciences and Research University, New Delhi, India; ^2^ Department of Pharmaceutics, College of Pharmacy, Jazan University, Jazan, Saudi Arabia; ^3^ Department of Pharmaceutical Chemistry, College of Pharmacy, Jazan University, Jazan, Saudi Arabia; ^4^ Department of Clinical Pharmacy, College of Pharmacy, Jazan University, Jazan, Saudi Arabia

**Keywords:** breast cancer, anticancer drugs, synthetic, phytochemicals, nanocarriers, drug delivery

## Abstract

Breast cancer being one of the most frequent cancers in women accounts for almost a quarter of all cancer cases. Early and late-stage breast cancer outcomes have improved dramatically, with considerable gains in overall survival rate and disease-free state. However, the current therapy of breast cancer suffers from drug resistance leading to relapse and recurrence of the disease. Also, the currently used synthetic and natural agents have bioavailability issues which limit their use. Recently, nanocarriers-assisted delivery of synthetic and natural anticancer drugs has been introduced to the breast cancer therapy which alienates the limitations associated with the current therapy to a great extent. Significant progress has lately been made in the realm of nanotechnology, which proved to be vital in the fight against drug resistance. Nanotechnology has been successfully applied in the effective and improved therapy of different forms of breast cancer including invasive, non-invasive as well as triple negative breast cancer (TNBC), etc. This review presents a comprehensive overview of various nanoformulations prepared for the improved delivery of synthetic and natural anticancer drugs alone or in combination showing better efficacy and pharmacokinetics. In addition to this, various ongoing and completed clinical studies and patents granted on nanotechnology-based breast cancer drug delivery are also reviewed.

## 1 Introduction

Breast cancer is the most common form of cancers amongst all types with 2.3 million cases diagnosed annually (Figueroa et al., 2021). Breast cancer-related mortality in women in the United States alone accounts for about 41,000 per year, or 15% of all cancer-related deaths (Liang et al., 2020). Early detection and extensive treatment techniques has decreased the cancer mortality in the last 2 decades improving the prognosis of breast cancer patients (Ganz and Goodwin, 2015). Considerable advancements have been made in the screening, diagnosis and treatment options for breast cancer (Fahad Ullah, 2019). Breast cancer starts with physiologically and molecularly diverse conditions in the breast, risk factors varying according to the type and genetic predisposition. Most notably mutations in the BRCA1 or BRCA2 genes remain significant component (Feng et al., 2018).

Treating breast cancer using estrogen receptor (ER) antagonists is the most established molecular targeted therapy approach as compared to other receptors (Sharma et al., 2018; Damodaran and Hortobagyi, 2021). Furthermore, the widespread use of tamoxifen, a selective ER modulator in treating breast cancer has been attributed with considerable advances in cure rates, quality of life, and preventing disease over the past quarter-century. Alternative therapies are also available for estrogen-dependent breast cancer which includes the use of aromatase inhibitors (AI) (anastrozole, letrozole, and exemestane) that impede the biosynthesis of androgens in tumor by down-regulating the aromatase enzyme (Chumsri et al., 2011). This results in decreased estrogen levels in tumor cells. Combination of receptor targets with chemotherapy at the molecular level have also been a significant step forward in the breast cancer treatment. Some of these combination targets include human epidermal growth factor receptor-2 (HER2) and the vascular endothelial growth factor (VEGF), which are used in tandem with chemotherapy drugs such as trastuzumab and bevacizumab (Munagala et al., 2011; Toss and Cristofanilli, 2015). In addition, inhibitors of downstream pathways such as PI3K/AKT/mTOR and RAS/MEK/ERK have the potential to be used therapeutically in some cases. There have also been reports of other tyrosine kinases being involved in cancer cell cancer progression and metastasis, such as insulin-like growth factor receptor (IGFR), poly-ADP ribose polymerase (PARP), and matrix metalloproteinases (MMP) inhibitors (Masoud and Pagès, 2017a). Several of these targeted medications showed significant improvements in the survival and prognosis of breast cancer patients. The MMP inhibitors work by blocking the activity of MMPs, a family of enzymes involved in the breakdown of extracellular matrix (ECM) proteins. ECM breakdown is an important step in cancer invasion and metastasis. By inhibiting MMPs, MMP inhibitors may prevent cancer cells from invading and spreading to other parts of the body. These MMP inhibitors include Batimastat, Marimastat, Prinomastat, BAY 12–9,566, Tanomastat, etc., The PARP inhibitors work by blocking the activity of poly (ADP-ribose)polymerase (PARP), an enzyme involved in DNA repair. PARP inhibitors specifically target cancer cells that have mutations in the BRCA1 and BRCA2 genes, which are involved in DNA repair. By inhibiting PARP, these cells are unable to repair DNA damage, leading to cell death. Some examples of drugs acting as PARP inhibitors are Olaparib, Rucaparib, Niraparib, Talazoparib, Veliparib, etc.

Significant advancements were made recently in the research aimed at targeting the molecular pathways of breast cancer, which have helped in understanding the targets at molecular level and developing targeted therapies to tackle it. Identifying and blocking the pathways that promote or perpetuate the proliferation and invasion of breast carcinoma cells is required for long-term efficacy in treating breast cancer. Over the past 2 decades, various monoclonal antibodies and small-molecule inhibitors have been proposed in clinical trials for their anticancer properties (Schlotter et al., 2008). Breast cancer has several molecular target pathways, including the EGFR Family, the vascular endothelial growth factor (VEGF) family, the RAS/MEK/ERK Pathway, cell cycle and apoptosis, estrogen receptor (ER) antagonist, targeting invasion and metastasis, insulin-like growth factor inhibitors, and the steroid hormone receptor.

However, the existing breast cancer therapy suffers from various issues including recurrence and relapse. The recurrence and relapse of breast cancer is mainly due to the resistance to conventional chemotherapeutic drugs and it is the leading cause of mortality despite significant research breakthroughs in the breast cancer therapy (Gote et al., 2021). Pharmacokinetics and metabolism of the tumor changes by the drug resistance and tumor protein P53, ATP-binding cassette (ABC), microtubules (MT), permeability glycoprotein (P-gp), HER2, topoisomerase, and breast cancer type 1 (BRAC1) lead to drug resistance in various ways. It also affects the mitochondrial metabolism, fatty acid synthesis, redox metabolism, and glycolysis in the breast cancer cells. All these resistance pathways are induced by the drugs including lapatinib, trastuzumab, adriamycin, paclitaxel, and tamoxifen (Gonzalez-Angulo et al., 2007). Owing to the fact that tumors contain distinct cancer-causing genes which frequently undergo several mutations, modified treatments can be achieved by identifying innovative multi-target agents and combining them. Chemotherapeutic drugs given intravenously result in widespread systemic dispersion and drug toxicity in cancer cells (Vasan et al., 2019). As a result, therapeutic medications that target non-cancerous cells and tumor locations are unable to provide adequate drug dose, resulting in poor therapy. Furthermore, the existing treatment is beset by low solubility and bioavailability of drugs at cancer locations. Numerous techniques are being adopted in order to overcome the drug resistance including application of nanotechnology, development of novel synthetic analogues of currently used drugs, repurposing drugs, combination of drugs, immunotherapy, patient monitoring, and synthetic lethality. Among these techniques, application of nanotechnology in preparing the nanoformulations of existing synthetic and natural anticancer molecules has garnered much attention and significant advancements have been made in this field.

Nanotechnology is one of the fastest growing fields of science and technology in the world; and it is frequently applied in breast cancer theranostics. As time progresses, nanotechnology aids in the discovery of novel research methodologies in oncology, even at the molecular level. Nanoparticles are unique in different ways and they can be used as nanomedicine in therapeutic applications. This review focuses on the recent studies aimed to enhance the targeted delivery of anticancer molecules of synthetic and natural origin using nanotechnology and its role in combating the drug resistance. Moreover, this article includes a research study based on the selection of an anticancer dose using combination index since drug combinations can have synergistic, antagonistic, or additive effects. Using the combination index, the dose of a combination of natural and synthetic drug was determined on two breast cancer cell lines, MCF-7 and T47D.

## 2 Nanomaterials to combat drug resistance

Drug resistance has become a significant concern in cancer therapy as the number of cancer treatment options is growing day by day. Cancer progression and a bad prognosis are caused by multidrug resistance, which results in the failure of cancer therapy. The mechanisms underlying drug resistance are well understood now. Interstitial fluid pressure, overexpression of the ATP binding cassette (ABC) efflux transporters, faulty apoptotic machinery, and a hypoxic or acidic tumor microenvironment are some of the factors implicated in medication resistance (Yao et al., 2020). Nanotechnology-based drug delivery devices play a crucial role in the treatment of cancer patients who developed drug resistance to conventional treatments. Nanoparticles that target the processes involved in drug resistance may be able to improve their effectiveness (Mba and Nweze, 2021). Targeting efflux transporters, which are the members of ABC transporter family, also play a significant role in the development of drug resistance. This transporter causes the drug to be effluxed out of the cell, reducing the therapeutic effect. P-glycoprotein (P-gp) is the most important efflux transporter and is overexpressed in some drug-resistant cancers (Waghray and Zhang, 2018; Robinson and Tiriveedhi, 2020). Nanoparticles can assist in bypassing the medication through the efflux transporter in these circumstances and polymer based nanoparticles can inhibit P-gp and change the regulation of drug release (Peng et al., 2020).

It has been reported that the nanoparticles containing COX-2 inhibitors along with doxorubicin were able to combat the multidrug resistance of breast cancer cells (Zhang et al., 2019). Furthermore, by suppressing the ABC transporters expression, co-delivery of anticancer agents with P-gp-targeted si-RNA using nanoparticles were found to aid in the treatment of drug-resistant tumors (Mirzaei et al., 2022). It was revealed that the lipid-based nanoparticles containing surfactant and co-surfactant helped in the prevention of drug from being effluxed by P-gp (Mangla et al., 2020). Cancer cells that have malfunctioned apoptotic machinery are able to circumvent apoptosis and improve their survival leading to drug resistance. Discrepancies in the apoptotic pathway are frequently produced by de-regulation of the nuclear factor-kappa B (NF-κB) and the anti-apoptotic protein Bcl-2; both of which are overexpressed in many tumors (Rinkenbaugh and Baldwin, 2016; Singh et al., 2019a). The nanoparticle-based delivery system exhibited improved cytotoxicity by causing apoptosis via downregulation of NF-κB and Bcl-2 expression along with the suppression of efflux transporter expression, thereby promoting apoptosis (Haider et al., 2022). The upregulation of drug efflux proteins has been shown to be mediated by hypoxia. Hypoxia creates a oxygen gradient inside the tumor which increases the heterogeneity of tumor and promotes more aggressive phenotype in the tumor cell itself. A hypoxia-inducible factor (HIF-1) has been identified to be overexpressed in a number of human cancer forms and plays a key role in treatment of drug resistance (Jun et al., 2017). Another therapeutic technique to overcome drug resistance is to target HIF-1 and nanoparticles such as PEGylated or non-PEGylated liposomes carrying HIF-1 siRNA were found to assist in treating hypoxia and drug resistance in cancer cells (Hajizadeh et al., 2020).

## 3 Nanomaterials from synthetic molecules

The drug therapy of breast cancer is still suboptimal owing to various issues affecting the efficacies of the currently used synthetic drugs. Major challenges associated with the existing therapy include insufficient biodistribution of drugs in the body which further decreases in the tumor tissues (Senapati et al., 2018). Drugs are more distributed to the healthy tissues causing toxicity and less into the tumor causing decreased efficacy. The microenvironment of tumor and drug resistance is other reason responsible for problem in the conventional therapy (Mansoori et al., 2017). Nanotechnology can be applied to overcome some of these limitations as they offer larger surface area-to-volume ratio providing good opportunity to manipulate the surface properties of drugs (Navya et al., 2019).

**FIGURE 1:**
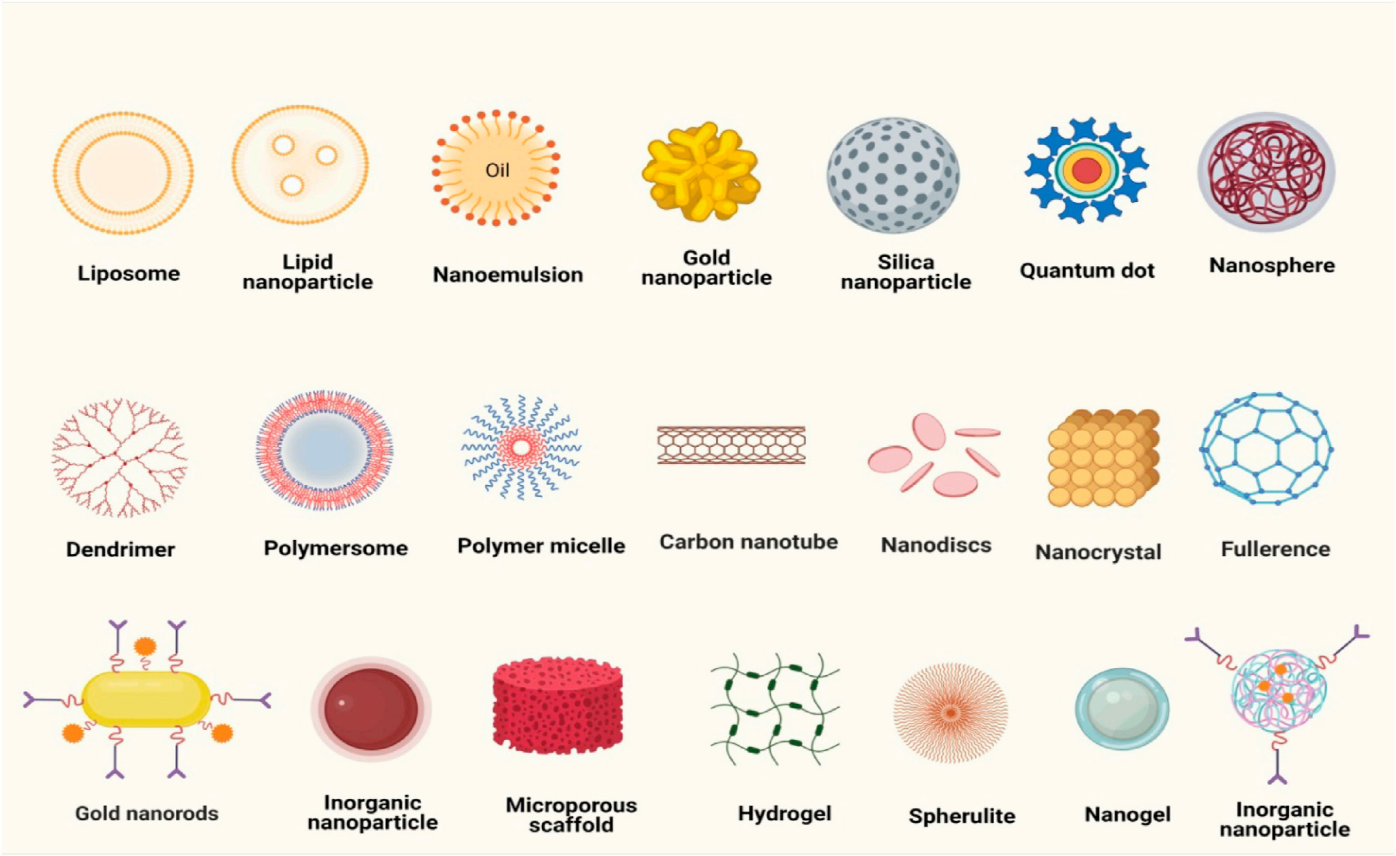
Various nanocarriers used for the delivery of synthetic anticancer drugs.

The fundamental goals of nanomedicine are to accurately diagnose and treat patients with minimal adverse effects; as well as to evaluate the efficiency of non-invasive therapeutic techniques (Longo et al., 2021). Nanomaterials used as carriers for the anticancer drugs utilized in the breast cancer treatment are broadly classified into two categories: organic and inorganic. Liposomes, micelles, dendrimers, and cyclodextrin are examples of organic materials; whereas inorganic materials include iron oxide, gold nanoparticles, and mesoporous silica nanoparticles (Gupta et al., 2021) (Figure 1).

**FIGURE 2:**
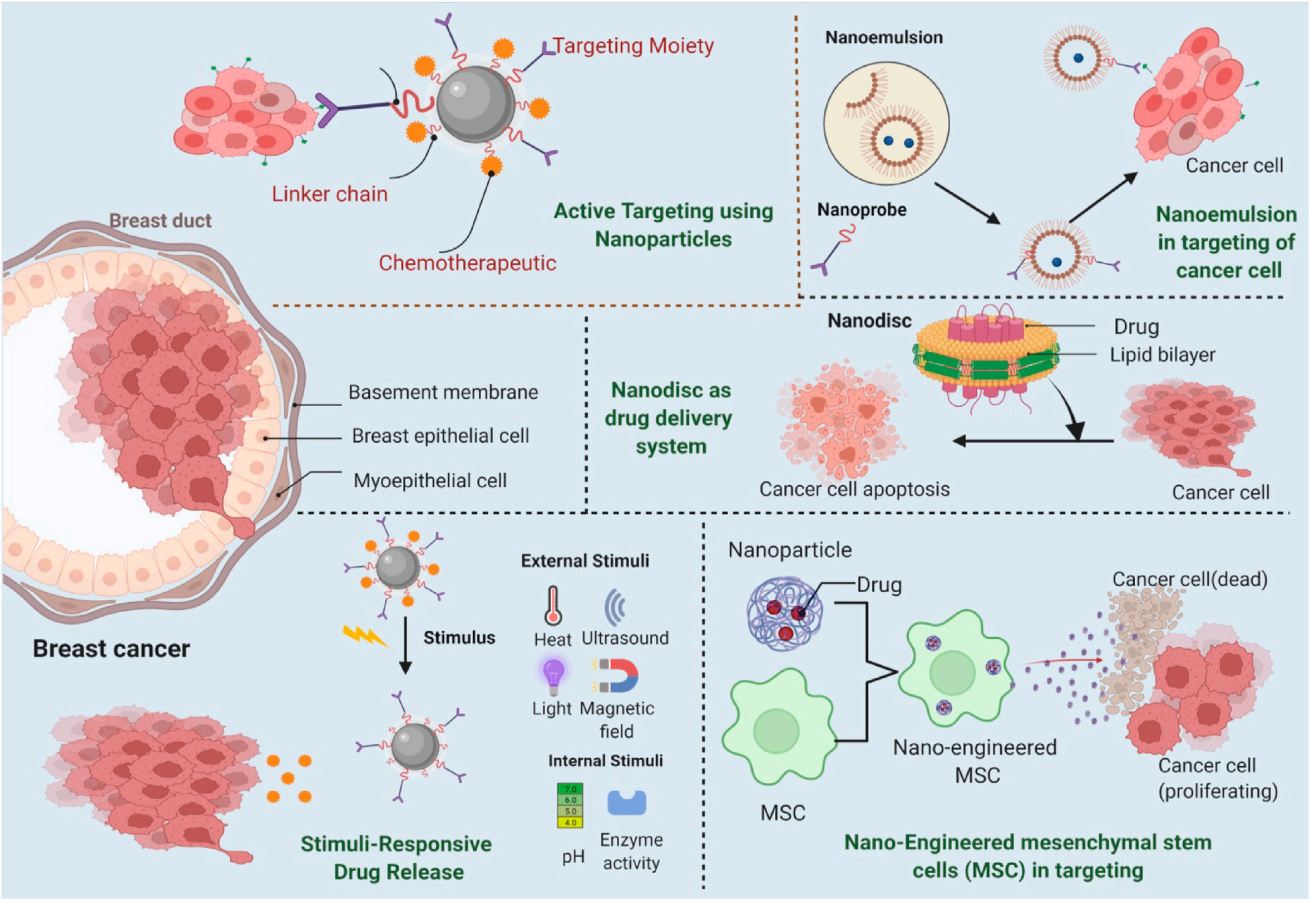
Mechanisms of nanocarriers as drug delivery system in breast cancer.

Nanotechnology can help achieve improved treatment in many ways including cancer targeting, increased endocytosis and extended circulation time which improves the access of anticancer drugs to the tumor sites (Figure 2). Moreover, encapsulating the drugs into the nanocarrier improves their solubility, stability and controlled release. Using nanocarriers, even the combination of drugs can be co-delivered to the target site in order to achieve synergistic anticancer activities. The magnetic nanoparticles on the other hand can also be utilized for the guided therapy, imaging as well as in drug delivery using external magnetic filed.

Numerous studies have been conducted on nanoparticles to assess their applications in breast cancer research. Some of the important nanocarriers used in breast cancer treatment as well as the dose and mode of administration are summarized in Table 1. These nanoformulations shown including NLCs, liposomes, phospholipid complexes, transferrin conjugates, solid lipid nanoparticles, β-cyclodextrin inclusion complexes, PEGylated liposomes, albumin-coated nanocrystals, cannabidiol-loaded microparticles, and chitosan-folate-coated mesoporous silica particles, have shown prominent effects against breast cancer cells. Synthetic drugs including exemestane, sulforaphane, tamoxifen, vitamin D3, etoposide and their combinations have been delivered using the nanoparticulate systems for effective breast cancer therapy.

**TABLE 1:** Synthetic drugs and their combinations delivered using nanotechnology for the treatment of breast cancer.

Drug (s)	Nanocarriers	Method of preparation	*In vitro*/*in vivo* studies	Cell lines	Dose of drug	Route of administration	Ref
Exemestene	NLCs	Ultra sonication	Particle size, Zeta potential, TEM, XRD, DSC, *In vivo* pharmacokinetic study	MCF-7	25 mg	Oral	Singh et al. (2019b)
Suforaphane and tamoxifen	NLCs	Ultrasonication	Particle size, Zeta potential, TEM, XRD, DSC, *In vivo* pharmacokinetic study, Acute toxicity		TAM: 5 mg/kg	Oral	Mangla et al. (2020)
					SFN: 15 mg/kg		
Exemestene	Liposomes	Thin film hydration	Particle size, Zeta potential, TEM, XRD, DSC, *In vivo* pharmacokinetic study	MCF-7	30 mg/kg	Oral	Singh et al. (2020)
Vitamin D3 + etoposide	Phospholipid complex	Solvent evaporation	Particle size, Zeta potential, TEM, FTIR, *In vivo* bio distribution study, *In vivo* anti-tumor efficacy study, Ttoxicity study	MDA MB-231	5 mg/kg	Intravenous	Agwa et al. (2021)
				MCF-7	7.5mg//kg		
Docetaxel	Phospholipid complex	Solvent evaporation	*In vitro* characterization, Cellular uptake study	Caco-2		Oral	Wang et al. (2020)
Tamoxifen	Transferrin conjugate Solid lipid nanoparticles	Hot emulsification	Particle size, Zeta potential, XRD, TEM, *In vitro* drug release	MCF-7		IV	Bhagwat et al. (2020)
Genistein	β-Cyclodextrins inclusion complex	Solvent evaporation	XRD, IR, NMR	MCF-7			Zafar et al. (2022)
Doxorubicin + Umbelliprenin	PEGylated liposomes	Thin film	Zeta potential, Particle size, *In vitro* drug release	MCF-7, MDA-MB 231, BT-474	10–500 μg		Gkionis et al. (2020)
Carfilzomib	Albumin coated nano crystals	Film hydration	Particle size, Zeta potential, XRD, TEM	MDA-MB-231, MCF-7, HCC 1943, and HCC1937	3 mg/kg	IV	Park et al. (2019)
Paclitaxel +	Cannabidiol loaded micro particles	Homogenization	Particle sixe, Zeta potential, XRD, TEM	MCF-7 and MDA-MB-231	3.65 mg/mL	IV	Fraguas-Sánchez et al. (2020)
Doxorubicin							
Anastrozole	Chitosan-folate coated mesoporous silica nanoparticles	Ultra sonication	Particle size, Zeta potential, XRD, SAXS, FTIR	MCF-7	1 mg/kg	IV	Bhavsar et al. (2019)

One of the synthetic drugs, Exemestane (EXE) which is a steroidal aromatase inhibitor and has been approved for the breast cancer therapy, was incorporated into NLCs as the drug encounters from the problem of limited water solubility and therefore, lesser oral bioavailability (Singh et al., 2019b). The EXE-NLCs showed extended release of the drug for upto 24 h *in vitro* and an augmented bioavailability by 3.9 folds was observed. Also, the permeation across the gut wall was shown to be improved as compared to the plain drug suspension. In an effort to explore the effects of combination of synthetic and natural molecules, tamoxifen (TAM) was loaded with sulforaphane (SFN) into a NLC system with the aim to enhance their oral delivery and to reduce the toxicity of TAM using SFN (Mangla et al., 2020). Formulation into NLCs was shown to enhance the intestinal permeability of TAM and SFN resulting into augmented oral bioavailability of TAM and SFN by 5.2 and 4.8 folds, respectively. Liposomes have been proved to be one of the most successful nanocarriers for the oral delivery of anticancer drugs; however, the harsh GI environment poses a challenge for effective drug delivery using liposomes. PEGylation has been shown to protect these liposomes for getting degraded in the GI and improves the oral drug delivery of drugs. In one such study, EXE was loaded into PEGylated liposomes and the stability in simulated gastric fluid was assessed and compared with conventional liposomes (Singh et al., 2020). PEGylation was reported to protect the liposomes from acidic degradation thereby improving the intracellular uptake of EXE as compared to the plain liposomes.

Similarly, the synergistic combination of vitamin D3/phospholipid complex and the anticancer drug etoposide (ETP) was explored by preparing vitaminD3/phospholipid decorated ETP-loaded nanomicelles (Agwa et al., 2021). This study aimed to target the drug into breast cancer cell lines using vit. D3 receptors which are overexpressed on breast cancer cells. The results showed improved anticancer activity of ETP on the tested cell lines without any toxicity to the lung fibroblasts demonstrating good selectivity of the prepared decorated nanomicelles. The use of docetaxel (DTX), one of the very useful drugs for breast cancer, is limited owing to its low solubility and permeability; therefore, DTX-phospholipid complex (DTX@PLC) was prepared and loaded into a self-microemulsifying drug delivery system (SMEDDS) (Wang et al., 2020). The DTX@PLC-SME increased the surface area and therefore the dissolution and permeability of DTX owing to the formation of a microemulsion in the gastrointestinal fluid. The action of drug TAM citrate was targeted to breast cancer cells in a strategy where TAM-loaded transferrin-conjugated-solid lipid nanoparticles (SLNs) were prepared with the aim to reduce its toxicity to uterus and other vital organs (Bhagwat et al., 2020). Results revealed improved cytotoxicity of TAM in a time- and concentration-dependent manner on MCF-7 cells as compared to the plain TAM solution.

A ternary inclusion complex of Genistein (GT) was prepared using β-cylcodextrin (β-CD) as complexing agent, while *D*-α-tocopherol-polyethylene glycol 1,000 succinate (TPGS) as ternary substance (Zafar et al., 2022). The prepared complex showed remarkable solubility enhancement as well as increased antioxidant and cell viability activity *in vitro* against human breast cancer cell lines MCF-7 in comparison to the pure GT. Similarly, the combination of doxorubicin (DOX) and umbelliprenin was loaded to PEGylated DSPC liposomes using microfluidic and thin-film methods and compared (Gkionis et al., 2020). The former technique produced particles with more homogeneous shape and size and an improved cytotoxicity against breast cancer cell lines. The poor aqueous solubility and stability of carfilzomib (CFZ) was addressed by preparing its nanocrystals which were subsequently coated with albumin to achive enhanced formulation stability and targeted drug delivery (Park et al., 2019). Improved anticancer activity of the developed formulation was observed with enhanced metabolic stability and improved cellular uptake. Therefore, encapsulation into the nanoparticles has proved to be an effective strategy in order to improve the biopharmaceutical properties of drugs and their combinations; and a sustained release of drug molecules was obtained over a long period of time.

## 4 Nanomaterials from natural molecules

Traditional medicines play an important role in many healthcare systems across the world, particularly in developing countries. According to a World Health Organization (WHO) estimate, traditional medicine covers the essential health requirements of around 80% of the population in impoverished countries, which is increasing day by day (World Health Organization, 2013). Natural anticancer medications are in increasing demand because they are efficient cancer cell inhibitors. Herb refers to a plant or their derived component that is used for its scent, flavor, and/or therapeutic properties, as well as for other purposes (El-Sayed and Youssef, 2019). Conventional medicinal herbs are naturally occurring plant-derived medications that are used to treat various types of diseases including cancer in local or regional healing traditions for hundreds of years with little or no chemical change to their formulation (Mahomoodally, 2013; Li et al., 2018). However, many natural molecules suffer from biopharmaceutical issues including poor solubility, low bioavailability, instability and poor permeability (Patra et al., 2018; Kesharwani and Bhat, 2020). Numerous novel drug delivery systems have been developed during the last 2 decades to function as carriers for a variety of bioactive chemicals or herbs; with the primary aim of increasing their bioavailability, reducing adverse effects, and avoiding drug degradation (Patra et al., 2018; Yap et al., 2021; Chavda et al., 2022). Moreover, these delivery systems improve the therapeutic efficacy, enhance stability and target the drugs to specific site and prevent first pass metabolism.

Treatment of breast cancer is undergoing extensive progress majorly with targeted therapies bypassing the severe side-effects of conventional chemotherapy (Masoud and Pagès, 2017b; Bukhari, 2022). The effectiveness of chemotherapy is restricted by the cancer cells’ resistance. As alternatives, phytochemicals and other natural molecules provide promising moieties for breast cancer prevention, treatment and therapy of difficult-to-treat mammary malignancies (Israel et al., 2018; Gregoriou et al., 2021). Phytochemicals significantly decrease the risk of cancer spread by direct tumor growth inhibition, apoptosis induction, and inhibition of tumor metastasis. Smart therapeutic approaches are the need of the hour for the treatment of various cancer types (Senapati et al., 2018). Numerous plant-derived phytochemicals have been found to have anticancer potentials and nanotechnological approaches of bioavailability enhancement are gaining rapid interest due to their tremendous potentials (Khan and Gurav, 2018; Dhupal and Chowdhury, 2020; More et al., 2021).

Niosomes, solid lipid nanoparticles, silver nanoparticles, gold nanoparticles, polymeric nanoparticles, liposomes, nanocapsules, and nanospheres are some of the delivery systems available for delivery of plant-based drugs including curcumin, cordycepin, balanocarpol, diallyl disulphite, epigallocatechin, gallic acid, sulphoraphane, resveratrol, punicalagin, ursolic acid, transferrin, bleomycin, and noscapine. Important phytochemicals showing anti-breast cancer activity and their corresponding developed nanoformulations are given in Table 2. These phytochemicals are found to have good efficacy in the treatment of breast cancer through various mechanisms. They are reported to mediate several physiological processes such as cellular uptake, pro-apoptotic activity, cytotoxicity, intracellular ROS generation, Bad, Bax, Caspase 3 and 9 protein level, nuclear shrinkage, stability, morphological change, cell migration, antioxidant activity, growth inhibitory activity, MMP-9 gene expression, etc. In contrast, they also decrease organ toxicities, Bcl-2 protein level, cell migration, P13K, pAKT level, pmTOR protein, cell viability, etc.

**TABLE 2:** Phytochemicals-based nanoformulations for the treatment of breast cancer.

Phytochemicals	Developed nanoformulation	Tested cell lines	References
Balanocarpol	Niosomes	ZR-75	Obeid et al. (2020)
Cordycepin	PLGA nanoparticles	MCF-7	Marslin et al. (2020)
Curcumin	Nanoliposmes/Dendrosomes sodium alginate nanoparticles/chitosan coated nanoliposomes/PVA cellulose nanocrystal hydrogels	4-T1/MCF-7	Hasan et al. (2014), Farhangi et al. (2015), Hasan et al. (2020), Hussein et al. (2021)
Diallyl disulphite	Solid lipid nanoparticles/Anti RAGE antibody conjugated solid lipid nanoparticles	MCF-7/MDA-MB-231	Talluri et al. (2016), Talluri et al. (2017)
Epigallocatechin	Solid lipid nanoparticles/Bombesin conjugated solid lipid nanoparticles	MDA-MB-231	Radhakrishnan et al. (2016), Radhakrishnan et al. (2019)
Gallic acid	Gum Arabic stabilized nanoparticles	Mcf/MDA-MB-231	Hassani et al. (2020)
Sulphoraphane	Pegylated nanoparticles/Iron oxide gold nanoparticles/Nanolipid carrier system	SKBR3/MCF-7/t47d	Danafar et al. (2017a)
Resveratrol	TPGS-Resveratrol-Solid Lipid Nanoparticles	SKBR3/PR cells	Wang et al. (2021)
Punicalagin	PGA/PEG nanoparticles	MCF-7	Shirode et al. (2015)
Ursolic acid	Liposomes	MDA-MB-231	Caldeira de Araújo Lopes et al. (2013)
Transferrin	Solid Lipid Nanoparticles	MCF-7	Bhagwat et al. (2020)
Bleomycin (BLM)	Gold nanoparticles	MDA-MB-231	Yang et al. (2016)
Artemisia Absinthium	Polymeric NPs	MCF-7/MDA-MB-231	Mughees et al. (2020)
Noscapine	Nanoparticles by pH coacervation technique	SK-BR-3	Sebak et al. (2010)

Various phytochemicals and plant extracts showing promising activities against breast cancer cell lines were further incorporated into nano-carriers in order to improve their activity and to combat their bioavailability issues. Metal-based nanoparticles (NPs) have shown considerable potential and a number of studies were reported where silver and gold nanoparticles of phytochemical compounds and plant extracts were prepared and tested *in vitro* against breast cancer cell lines. Silver and gold nanoparticles are noble nanoparticles with optimized shapes and sizes which were found to be effective as carriers in the cancer therapy.

Previously, silver nanoparticles (Ag-NPs) of *Agrimoniae herba* extract exhibited strong antineoplastic effect on A549 cells, indicating better antitumor effects than the extract alone (Qu et al., 2014). Similarly, the Ag-NPs of *Beta vulgaris* extract were also prepared and tested against human breast cancer (MCF-7), pharynx (Hep-2) and lung (A549) cancer cell lines (Venugopal et al., 2017a) and the results showed improved permeability and efficacy. In a similar study, Ag-NPs of *Syzygium aromaticum* extract showed *in vitro* cytotoxic activity against Hep-2 and MCF-7 cells and the results suggested its use as cancer therapeutics (Venugopal et al., 2017b). In yet another study, green tea, turmeric and garlic extracts-mediated Ag-NPs were prepared and characterized. Results revealed that the Ag-NPs prepared from turmeric extracts exhibited superior antioxidant and cytotoxic activity in comparison to other extracts on all four tested cancer cell lines including HeLa, MCF-7, A549 and Hep-2, *in vitro* (Arumai Selvan et al., 2018). Ag-NPs of *Jasminum officinale* L. leaves extract also showed high cytotoxicity against MCF-7 and -5,637 cells and an IC_50_ values of 13.09 μg/mL and 9.3 μg/mL, respectively were obtained, proving it to be an effective approach (Elhawary et al., 2020). Ag-NPs of *Cuminum cyminum* L. seed extract were also reported to posses anticancer activities when tested on MCF-7 and AU565 (human breast adenocarcinoma metastatic) cell lines (Dinparvar et al., 2020).

Similarly, gold NPs (Au-NPs) are gold particles of nanosized range (1–100 nm) which are being used in many studies as carriers for delivering anticancer agents of natural origin (Singh et al., 2021). In one such study, Au-NPs of *Nerium oleander* were prepared, characterized and anticancer potential was tested against MCF-7 cells. Results showed that the prepared Au-NPs induced apoptosis in the tested cell lines selectively (Barai et al., 2018). Recently, Au-NPs of phytochemicals of nano-ayurvedic drugs also called as “*Nano Swarna Bhasma*” were reported for the treatment of metastatic breast cancer (Khoobchandani et al., 2020). The characteristic features of Au-NPs of being biogenic and easy to synthesize, resulted in diverse research and applications. The anticancer phytocompound Withanolide-A conjugated with Au-NPs was also reported where the prepared nanoconjugate significantly induced SKBR3 cell growth blockage at half of the maximal-active concentration in comparison to the Withanolide-A alone (Tabassam et al., 2020).

Various other nanoformulations of phytochemicals were also prepared and screened against the human breast cancer cell lines such as niosomes, nanoemulsions, and nanovesicles. The nanoniosomes (a non-ionic surfactant based nanovesicles) of the phytochemical lawsone were fabricated by film-hydration method using non-ionic surfactants and cholesterol. The *in vitro* study exhibited significant antitumor efficacy of the nanoformulation against the MCF-7 cell lines in comparison to the Lawsone solution (Barani et al., 2018). In another study, diosgenin was loaded into niosomes in order to improve its solubility profile. The method adopted to prepare the niosomes was thin-film hydration technique and the cytotoxicity screening was performed on HepG2 cell line. The prepared niosomes showed good loading efficiency (89%) and the *in vitro* findings revealed that the niosomes exhibited enhanced anticancer effects 28.32% higher than the free diosgenin (Hajizadeh et al., 2019). Curcumin-loaded deformable nanovesicles were obtained by thin-film hydration technique followed by extrusion. Cytotoxicity studies were performed on MCF-7 cell lines using MTT assay technique. The prepared nanovesicles showed an IC_50_ of 20 μg/mL suggesting them to be successful candidates for the breast cancer therapy (Abdel-Hafez et al., 2018).

Resveratrol, a polyphenolic phytoalexin has significant pharmacological properties; however, it exhibits poor aqueous solubility issues. In lieu of its poor bioavailability issues, nanocarrier of resveratrol loaded NPs were synthesized by the emulsification method using Pluronic-F127 block copolymer and D-α-tocopheryl polyethylene glycol 1,000 succinate (Vitamin E-TPGS). The prepared NPs showed spherical shape, nanosize (179 ± 22 nm), good encapsulation efficiency (73% ± 0.9%) and optimum drug loading (6.2% ± 0.1%) and were reported to have remarkable efficacy against breast cancer cell lines (Gregoriou et al., 2021). The restricted use of anticancer phytochemical sulforaphane was successfully modified with the help of a reliable micellar drug delivery system using monomethoxy-poly (ethylene glycol)-poly (ε-caprolactone) (mPEG-PC) polymer. The anticancer efficacy studied on MCF-7 cells suggested their better efficacy against the breast cancer cell lines in comparison to pure sulforaphane (Danafar et al., 2017b). The nanoemulsion of Zataria essential oil with citrus-pectin was formulated and assessed for its anticancer potential. The resulting nanoemulsion enhanced the viability suppression of drug resistant breast cancer cell lines, MCF-7 and MDA-MB-231 as well as spheroids making it a viable and promising anti-proliferative candidate against breast cancer (Salehi et al., 2020a; Salehi et al., 2020b).

The phytosterol, Stigmasterol (STS)-loaded CD44 receptor targeted PEGylated nanohybrid phytoliposomes were prepared for synergistic chemotherapy along with doxorubicin (DOX) against the metastasis and proliferation of breast cancer cells. The nanocarriers were fabricated by thin-film hydration method and were tested both *in vivo* and *in vitro* against the breast cancer cell lines expressing various CD44 receptor levels. The PEGylated DOX-STS liposomes modified using hyaluronic acid (HA) were found to be promising carrier system towards CD44-overexpressing tumors (Gautam et al., 2020). In one such study, Thymoquinone (TQ)-loaded HA-conjugated Pluronic^®^-P123 and -F127 copolymeric NPs were prepared as drug loading vehicles for the delivery of anticancer phytochemical, TQ to TNBC cells. The TNBC is known to be the most non-responsive subtypes of all breast cancers towards the available therapeutic options (Bhattacharya et al., 2020).

On similar grounds, herbal anticancer phytochemicals can also be incorporated into novel nanocarrier systems such as a polymerosome system to achieve controlled release of natural anticancer agents with improved drug loading efficiency and controlled release from the nanocarriers (Nosrati et al., 2019). It was reported that the plant polyphenols or flavonoids exhibited properties that are important for the therapy of breast cancer. This can be attributed to their antioxidant and anticarcinogenic potential that assists in cell cycle arrest as well as suppression of the uncontrolled cell division in cancer cells, and the activity can further be enhanced by incorporating them into nanocarriers (Sindhu et al., 2021). The essential oils obtained from plants are also a big source of phytochemicals with myriad biological activities including anticancer. However, their use is still beset because of low bioavailability, and rapid degradation profile which can be improved using nanocarriers.

## 5 Nanomaterials targeting triple negative breast cancer (TNBC)

The term “triple negative breast cancer” (TNBC) refers to a form of breast cancer in which none of the breast cancer’s expression receptors—human epidermal growth factor receptor (EGFR), progesterone receptor (PR), and estrogen receptor (ER) are present. As per the gene expression profile analysis, TNBC is classified as the subtype of basal-like breast cancer (BLBC). The TNBC constitutes about 15%–25% of all breast cancers and has a more aggressive biology, including early stage metastatic disease, visceral organ metastases, highly progressing disease, extremely limited responses to existing treatment regimens, and overall shorter survival rate compared with other major breast cancer subtypes and the mortality rate is 40% within the first 5 years post diagnosis (Yin et al., 2020). In TNBC patients, the relapse period remains between 19 and 40 months, as compared to 35 and 67 months in case of non-TNBC patients. Approximately 75% of TNBC patients die within 3 months of the recurrence (Lyons, 2019). Also, it was reported that the risk of TNBC increased as the use of oral contraceptives increased in duration (Howard and Olopade, 2021).

In comparison to other cancer types, TNBC has limited treatment options. Therefore, chemotherapy has become the main approach of TNBC using the drugs Cisplatin, Docetaxel, Doxorubicin, Adriamycin, Taxan, Anthracycline, 5-Flurouracil and EGFR inhibitors. FDA has recently approved new drugs acting as PARP inhibitors which can destroy TNBC cancer cells (Barchiesi et al., 2021). Although, these chemotherapies are effective but are associated with critical biopharmaceutical issues. Therefore, to overcome these issues, use of nanocarriers was found to be a better alternative for delivering the chemotherapeutic drugs. In addition to serving as a delivery system, nanocarriers were found to be a highly successful therapeutic approach for TNBC (Nahvi et al., 2022). The circulation of drugs in blood is increased by nanoparticles, which also decreases the phagocytosis and reticuloendothelial system (RES) absorption. Moreover, it reduces the side effects associated with chemotherapy. Drug repurposing is currently in vogue along with chemotherapy for the treatment of TNBC. Repurposed drug include evofosfamide, azadiradione, niclosamide, diindolylmethane derivatives, ginsenosides, triptorelin, etc (Ávalos-Moreno et al., 2020). These repurposed medications are currently being administered for the TNBC treatment using a variety of nanocarrier systems. Different types of nanocarriers which showed effectiveness in TNBC include liposomes, lipid nanocarriers, micelle, dendrimers, carbon nanotubes, polymeric nanoparticles and gold nanoparticles. Divergent methods are utilized for the preparation of these nanocarriers which include solvent dispersion method, film hydration method, high speed homogenization, dissolution method, emulsification-solvent evaporation, high-pressure homogenizer, gel electrophoresis, chemical vapor deposition, self -assembled polymersomes and adhesion method (Krishnamoorthy and Mahalingam, 2015). Various nanocarriers used for the delivery of anticancer drugs used in chemotherapy targeting TNBC are presented in Table 3.

**TABLE 3:** Types of drugs and nanocarriers utilized in the treatment of TNBC.

Drug	Nanocarrier	Method of preparation	Study outcome	Ref
Evofosfamide	Chitosan oligosaccharide decorated liposomes	Solvent dispersion method	CO-HPPH-TH302/Liposome has extremely effective therapeutic strategy for CD44-overexpressing TNBC	Ding et al. (2021)
Azadiradione	Liposomes	Film hydration method	Drug blood circulation is increased by nanoparticles, which also decrease RES phagocytosis and uptake	El-Senduny et al. (2021)
Cisplatin	Lipid nanocarriers	Thin-film hydration method, high speed homogenization	Caspase-9 and p21 expression are both increased, and EGFR is inhibited	Andey et al. (2015)
Docetaxel	PEGylated lipid-core micelle	Dissolution method	Improved the antitumor effects and reduced the side-effects of docetaxel	Chen et al. (2021)
Niclosamide	Phenyl boronic acid-modified solid lipid nanoparticles	Emulsification-solvent evaporation	effective strategy to eradicate TNBC cells	SS Pindiprolu et al. (2020)
Niclosamide	Solid lipid nanoparticles	Emulsification-solvent evaporation	SLNs loaded niclosamide improved the anticancer efficacy against TNBC	Pindiprolu et al. (2019)
Diindolylmethane derivatives	Nano structured lipid carrier	High-pressure homogenizer	NLC of diindolylmethane derivatives was found to be effective in TNBC	Godugu et al. (2016)
Citral	Nano structured lipid carrier	High-pressure homogenization	Citral was identified to target TNBC with NLC as a feasible and efficient delivery system	Nordin et al. (2020)
siRNA	Dendrimers	Gel electrophoresis	Potential siRNA delivery vehicles using polyamidoamine dendrimers to treat TNBC	Jain et al. (2019)
Ginsenosides	Carbon nanotubes	Chemical vapor deposition	Carbon nnaotubes was found to be potential therapeutic strategy for immunotherapy of TNBC	Luo et al. (2021)
Doxorubicin	Polymeric nanoparticles	Self -assembled hypoxia-responsive polymersomes	Potential drug carriers for the treatment of TNBC.	Mamnoon et al. (2020)
Triptorelin	Gold nanoparticles	Adhesion method	Triptorelin-functionalized PEG-coated gold nanoparticles have therapeutic efficacy to treat TNBC cells	Uzonwanne et al. (2022)

## 6 Challenges associated with nanomaterials

There are several challenges in nanoparticulate developments that need to be addressed before these materials can be widely used in medicine. One of the main challenges is the potential toxicity of nanomaterials. Due to their tiny size, nanoparticles can enter cells and tissues, potentially causing harm. It is crucial to thoroughly assess the toxicity associated with nanoparticles and understand their effects on biological systems before they can be introduced to clinical settings. Another challenge is the complexity of nanoparticle manufacturing. The production of nanoparticles can be a complicated and expensive process, requiring specialized equipment and expertise.

The reproducibility and scalability of nanoparticle manufacturing also need to be addressed to ensure that they can be prepared at a larger scale for widespread use. The shelf-life and stability of nanoparticles can also be a challenge. Nanoparticles can be prone to aggregation, which can affect their properties and efficacy. Additionally, the long-term stability of nanoparticles needs to be evaluated to ensure that they maintain their properties over time. Finally, there are regulatory challenges associated with the use of nanomaterials in medicine. The regulatory landscape for nanoparticles is still evolving, and there are currently no specific guidelines for their use in clinical applications. This can create uncertainty for researchers and companies developing nanoparticulate therapies and may slow down their development and approval processes.

## 7 Advantages of nanomaterials over traditional therapies

The use of nanomaterials emerged as a potential approach in the field of drug delivery owing to their unique chemical, physical and biological properties. They offer several advantages over traditional therapies based on pharmacokinetics (PK), pharmacodynamics (PD), and therapeutic benefits as shown in Table 4.

**TABLE 4:** Advantages of nanomaterials over traditional therapy on the basis of PK, PD and therapeutic benefits.

Criteria	Nanomaterials	Traditional therapy
PK Advantages	Enhanced bioavailability and extended circulation time due to smaller size and surface area	Limited bioavailability and short circulation time
	Increased stability and solubility, and improved tissue penetration and accumulation	Limited stability and solubility, and poor tissue penetration and accumulation
	Limited capacity for drug encapsulation and combination, and lack of versatility for multifunctional therapeutics	Limited accumulation in tumor tissues and potential for off-target effects
	Ability to cross biological barriers such as the placenta, and potential for oral delivery	Limited ability to cross biological barriers such as the placenta, and poor bioavailability with oral delivery
PD Advantages	Increased specificity and targeted delivery to diseased cells/tissues due to surface modification and functionalization	Non-specific and systemic distribution
	Controlled release and sustained drug delivery, and ability to overcome biological barriers including the blood-brain barrier	Immediate release and rapid drug clearance, and difficulty in crossing the biological barriers
	Ability to target specific tumor markers and overcome multidrug resistance mechanisms, and potential for synergistic drug combinations	Non-specific and systemic distribution and potential for multidrug resistance and toxicity
	Reduced immunogenicity and potential for immunomodulatory effects, and ability to target immune cells	Increased immunogenicity and potential for adverse immune reactions, and limited ability to target immune cells
Therapeutic Benefits	Lower effective doses, reduced toxicity, and improved patient compliance	Higher effective doses, increased toxicity, and poor patient compliance
	Ability to encapsulate multiple drugs and synergistic drug combinations, and potential for multifunctional therapeutics	Limited capacity for drug encapsulation and combination, and lack of versatility for multifunctional therapeutics
	Potential for reducing tumor size and preventing metastasis, and ability to deliver both chemotherapy and targeted therapies	Limited efficacy in reducing tumor size and preventing metastasis, and potential for severe side effects and treatment resistance
	Potential for personalized medicine and tailoring therapies to individual patients’ needs, and ability to treat diseases at early stages	Limited ability to personalize therapies and treat diseases at early stages, and reliance on palliative care for certain diseases

## 8 PHOTOIMMUNONANOTHERAPY (PINT)

PhotoImmunoNanoTherapy (PINT) is a type of cancer treatment that combines three different approaches: phototherapy, immunotherapy, and nanotechnology. It involves the use of nanoparticles that are designed to target cancer cells and then activate them with light to destroy them. The nanoparticles are usually coated with antibodies that specifically bind to cancer cells, making them a highly targeted approach to treat cancer. Once the nanoparticles have been taken up by the cancer cells, light is used to activate them, causing the nanoparticles to release reactive oxygen species (ROS) that kill the cancer cells. In addition to the direct effects of the phototherapy, PINT also harnesses the power of the immune system to fight cancer. The ROS generated by the nanoparticles can also trigger an immune response that targets the cancer cells, further enhancing the effectiveness of the treatment. PINT is still in the early stages of development, but it has shown promising potential in preclinical studies and holds great potential as a new cancer therapy.

PINT offers several advantages in the treatment of breast cancer. It can be highly targeted, meaning that the nanoparticles can be designed to specifically bind to breast cancer cells only and spare the healthy cells. Secondly, PINT can be combined with other treatment approaches including chemotherapy or radiation therapy, to enhance their effectiveness. Third, PINT can activate the immune system to fight the cancer cells, providing a systemic response that can help prevent the cancer from spreading. Several preclinical studies have shown promising results for PINT in breast cancer. For example, in one study, researchers used PINT to target breast cancer cells in mice and found that the treatment was highly effective at reducing the size of tumors and preventing metastasis. Another study showed that PINT combined with immunotherapy was able to eliminate breast cancer cells in mice without causing any significant side effects.

## 9 Selection criteria for a nanoparticulate-based therapy

Nanoparticulate-based therapy is an upcoming field of medicine that involves the use of tiny particles for the targeted delivery of drugs or therapeutic agents to specific cells or tissues in the body. The selection of appropriate nanoparticles for therapy is crucial for the success of the treatment. The selection criteria for nanoparticulate-based therapy involve numerous factors such as size, surface charge, composition, and biocompatibility of the nanoparticles. The size of the nanoparticles is an important consideration because it determines their ability to penetrate biological barriers and reach the targeted cells or tissues. The ideal size for nanoparticles used in therapy is typically between 10 and 200 nm. Additionally, the surface charge of nanoparticles can also influence their ability to target specific cells or tissues. Particles with a neutral or slightly negative surface charge tend to be more stable and have longer circulation times in the bloodstream, which is critical for the delivery of drugs to target cells. The composition of nanoparticles also plays a crucial role in determining their suitability for therapy. For example, nanoparticles made of biodegradable or biocompatible materials such as lipids, proteins, or polysaccharides tend to have fewer adverse effects and can be easily metabolized and eliminated from the body.

Moreover, nanoparticles can be functionalized with specific ligands or targeting molecules that enable them to specifically bind to the target cells or tissues, improving their specificity and efficacy. Finally, the biocompatibility of nanoparticles is also essential for successful therapy. Nanoparticles should not trigger an immune response or cause toxicity or inflammation in the body. Hence, it is critical to carefully assess the toxicity and biocompatibility of nanoparticles in preclinical studies before using them in clinical trials. Overall, the selection of appropriate nanoparticles for therapy involves a careful balance between the size, surface charge, composition, and biocompatibility of the nanoparticles to ensure their safety and efficacy for therapeutic use.

## 10 Recent patents on nanomaterials for anticancer therapy

To comprehend the ongoing research into the breast cancer treatment using nanocarriers and nanotechnology, an effective search was conducted using the databases ‘Patentscope’, ‘Espacenet’, ‘USPTO public pair’, ‘Google patents’ and ‘InPASS’, to identify the relevant patents in this domain, which yielded a total of 3,239 results. To attain an accurate sequence of ongoing research work, the results obtained were further restricted to recently granted patents in the year 2021 and 2022. Consequently, some of the relevant inventions were studied, and the most pertinent results were included in the current review to facilitate comprehension of the research work. These studies should provide a conceptual framework for conducting more programmed and effective breast cancer research and implementing nanotechnology-based strategies. Table 5 provided below summarizes some of the selected inventions.

**TABLE 5:** Recent patents granted on nanotechnology-based treatment of breast cancer.

Application/Publication number	Title of invention	Summary of invention	Ref
AU2022200881A1	Mesoporous Silica Nanoparticules with Lipid Bilayer Coating for Cargo Delivery	This invention provided a nanocarrier consisting of a silica body with pores, coated with a lipid bilayer containing a cargo-trapping agent and pores are sealed by the phospholipid bilayer	Liu et al. (2022)
JP2022058656A	A *Micrococcus* genus bacteria origin nano vesicle and its use	The current invention provided an improved composition for cancer management	Kim (2022)
CN114259477A	A nano delivery system for promoting penetration, relieving tumour hypoxia, and targeting tumour cell and preparation method and application thereof	The present invention provided unique and intelligent nanodelivery system for targeted drug delivery with enhanced penetration and reduced toxicity	Shen et al. (1142)
WO/2021/046480	Nucleic acid-mediated delivery of therapeutics	This invention involved the compositions of therapeutic compounds complexed with nucleic acid fragments to form nanoparticles, along with associated small molecules, and their use	Kwon et al. (2021)
WO/2021/011501	Therapeutic Constructs for co-delivery of mitotic kinase inhibitor and immune checkpoint inhibitor	The disclosure describes therapeutic constructs consisting of a delivery particle, immune checkpoint inhibitor, mitotic kinase inhibitor, and chemical linker. These constructs can treat different cancer types, improve drug delivery, and induce cancer cell death via therapeutic and immune effects	Yantasee et al. (2021)
US20220117894A1	Targeted Polymerized Nanoparticles for Cancer Treatment	The invention relates to a novel drug delivery system comprising hybrid liposomal nanoparticles with polymerizable and non-polymerizable lipids, used to deliver therapeutic agents and target specific diseases	Nagy et al. (2022)
AU2021290200A1	Biomarkers For Nanoparticle Compositions	This invention provided nanoparticles containing an mTOR inhibitor and albumin for the treatment of diseases such as cancer, restenosis, and pulmonary hypertension based on the presence or absence of an mTOR-activating aberration	Desai (2021)
AU2021269331A1	A pharmaceutical composition comprising stable, amorphous hybrid nanoparticles of atleast one protein kinase inhibitor and atleast one polymeric stabilizing and matrix-forming component	This invention was related to protein kinase inhibitor- and matrix-forming polymeric hybrid nanoparticles that were stable and amorphous and could be used in pharmaceutical compositions to treat diseases	Brisander et al. (2021)
CN114306628A	A two-dimensional black phosphorus hybrid nano-drug targeting HER2 positive breast cancer modified based on PEG-MAL	This invention involved hybrid nanoparticles using PEG-MAL-modified 2D black phosphorus for targeted photothermal immune composite therapy of HER2-positive breast cancer with higher drug loading and photothermal conversion rates	Benqing and Jinxing (1143)
CN114272209A	A nano-lipid microsphere of chemical anti-tumour medicine, and combined application of the nano-lipid microsphere and tumour immunization therapy medicine	A novel approach to treat cancer wass provided by this invention, which utilized the nano-lipid microspheres containing anticancer drugs combined with tumor immunotherapeutic drugs such as anti-PD-1 monoclonal antibody (alpha PD-1) in three different treatment schemes based on the *in vivo* effects. These combinations selectively released the anticancer drugs at the tumor site	Qingpo et al. (1142)
CN114191549A	Docetaxel-curcumin solid lipid nano-particle targeted by intestinal SGLT1 and preparation method and application thereof	The invention based on the targeted docetaxel-curcumin solid lipid nanoparticle that combines chemotherapy and photodynamic therapy to effectively treat tumors, with a preparation method and application provided to solve issues of adverse reactions and low bioavailability of the drugs	Gang et al. (1141)
CN114099656A	A co-loaded nano preparation based on antigen and active polysaccharide and preparation method and application thereof	The invention discloses a co-loading of antigen and active polysaccharide in nanocarrier that showed more effective and desired tumor immunotherapy effect	Conghui (1140)
CN114010786A	A method for treating triple-negative breast cancer by double antisense nucleic acid synergetic photodynamic therapy	Using double antisense oligonucleotide synergistic photodynamic therapy, the inventors created a nanodelivery system for the treatment of TNBC that successfully induced apoptosis in tumor cells	Zhaoqi et al. (1140)

## 11 Clinical studies on anticancer nanomaterials

Chemotherapy, surgery, hormone therapy, radiation, and targeted therapy are the cornerstones of breast cancer treatment. However, scientists continue to investigate new treatments and medications, as well as novel combinations of existing treatments. Participants in clinical trials have the opportunity to receive a new, potentially superior treatment and to contribute to cancer research that may improve the breast cancer treatment for others. However, there is a new emphasis on adding targeted therapies to hormone therapy for HR-positive cancers that are advanced or metastatic. These treatments could delay the need for chemotherapy and ideally, increase survival. The United States Food and Drug Administration (USFDA) approved the use of palbociclib, ribociclib, and everolimus in conjunction with hormone therapy to treat advanced or metastatic breast cancer. It has been demonstrated that ribociclib prolongs the lives of patients with metastatic breast cancer. Abemaciclib can be used with or after hormone therapy to treat HR-positive, HER2-negative advanced or metastatic breast cancer. table 6 showed list of drugs approved by the USFDA from 2020 to 2022.

**TABLE 6:** List of the drugs approved by USFDA from 2020–2022 for advanced or metastatic breast cancer.

Drug molecule	Brand name/Manufacturer	Approval date	Remarks	Ref
Fam-trastuzumab deruxtecan-nxki	Enhertu/Daiichi Sankyo, Inc	05 August 2022	Adult patients with HER2-low (IHC 1+ or IHC 2+/ISH) metastatic or unresectable breast cancer who previously had chemotherapy in a metastatic context or who have experienced a disease recurrence while receiving adjuvant chemotherapy or within 6 months of finishing it	(fda.gova)
Olaparib	Lynparza/AstraZeneca Pharmaceuticals, LP	11 March 2022	Adults with deleterious or suspected detrimental germline-BRCA mutations (gBRCAm) who received neoadjuvant or adjuvant chemotherapy for high risk early breast cancer	(fda.govb)
Abemaciclib	Verzenio/Eli Lilly and Co.	12 October 2021	For the adjuvant treatment of adults with HR-positive, HER2-negative, node-positive, early breast cancer with a high risk of recurrence and a 20% Ki-67 score, as determined by an FDA-approved test, a combination of abemaciclib and endocrine therapy (tamoxifen or an aromatase inhibitor) was advised. This was the first CDK-4/6 inhibitor approved for adjuvant treatment of breast cancer	(fda.govc)
Pembrolizumab	Keytruda/Merck	26 July 2021	The FDA has approved pembrolizumab as a single agent adjuvant treatment following surgery and as a neoadjuvant treatment for high-risk, early-stage TNBC in combination with chemotherapy	(fda.govd)
Sacituzumab govitecan	Trodelvy/Immunomedics Inc	7 April 2021	Sacituzumab govitecan received FDA approval for patients having unresectable locally advanced or metastatic-TNBC (mTNBC) who had received two or more previous systemic therapies, including at least one for metastatic cancer	(fda.gove)
Margetuximab-cmkb	Margenza/MacroGenics	16 December 2020	For the treatment of metastatic HER2-positive breast cancer in people who had received two or more prior anti-HER2 regimens, at least one of which was for metastatic cancer, the FDA approved margetuximab-cmkb (MARGENZA, MacroGenics) in combination with chemotherapy	(fda.govf)
Ppembrolizumab	Keytruda/Merck & Co.	13 November 2020	The FDA had approved the use of pembrolizumab in combination with chemotherapy to treat patients with locally recurrent, incurable, or metastatic-TNBC, whose tumors expressed PD-L1 (CPS 10), as determined by an FDA-approved test	(fda.govg)

## 12. Conclusions and future Directions

As the molecular biology and etiology of breast cancer is understood well, significant progress have been achieved in the recent years in the diagnosis and treatment of this disease. Various molecular targets have been identified and several drugs are developed showing good potential in managing the disease. The limitations of currently used drugs have also been successfully overcome by developing the nanomedicines using the nanotechnology. Incorporating the drugs and their combinations in nanocarriers has shown better potential as far as the biopharmaceutical issues of the drugs are concerned. Nanoparticles have emerged as versatile drug carrier systems which allow delivery of multiple anticancer agents with the ability to target more than one type of cancer leading to advancements in both diagnosis and treatment of cancer. However, adopting these advanced strategies should be exercised with caution owing to the complexity of the nanoformulations which might lead to immunogenicity and toxicity. Also, the higher production cost of nanomedicines and limited upscalability are crucial factors with the manufacturing of nanomedicines which need to be addressed. Nanocarriers are also associated with some drawbacks including the possible immunogenicity, toxicity and fast excretion. The solid-lipid nanoparticles (SLNs) have low drug loading capacities and contain other complex colloidal structures. The cationic lipids present in liposomes can cause toxicity and also suffer from rapid degradation in body. Till date, only few nanomedicines have received the approval from USFDA out of which Doxil and Abraxane are the two most successful ones for the breast cancer treatment in clinical settings. Several other promising nanocarriers-assisted targeted delivery of synthetic and natural molecules are being investigated in recent years and a number of other formulations are in clinical trial stage.
